# Comparative Transcriptional Profiling of Two Contrasting Barley Genotypes under Salinity Stress during the Seedling Stage

**DOI:** 10.1155/2013/972852

**Published:** 2013-05-19

**Authors:** Runhong Gao, Ke Duan, Guimei Guo, Zhizhao Du, Zhiwei Chen, Liang Li, Ting He, Ruiju Lu, Jianhua Huang

**Affiliations:** ^1^Biotech Research Institute, Shanghai Academy of Agricultural Sciences, Beidi Road 2901, Minhang District, Shanghai 201106, China; ^2^Shanghai Key Laboratory of Agricultural Genetics and Breeding, Shanghai 201106, China; ^3^College of Fishery and Life Science, Shanghai Ocean University, Shanghai 201306, China

## Abstract

Salinity is one of the major abiotic stresses that affect crop productivity. Identification of the potential novel genes responsible for salt tolerance in barley will contribute to understanding the molecular mechanism of barley responses to salt stress. We compared changes in transcriptome between Hua 11 (a salt-tolerant genotype) and Hua 30 (a salt sensitive genotype) in response to salt stress at the seedling stage using barley cDNA microarrays. In total, 557 and 247 salt-responsive genes were expressed exclusively in the shoot and root tissue of the salt-tolerant genotype, respectively. Among these genes, a number of signal-related genes, transcription factors and compatible solutes were identified and some of these genes were carefully discussed. Notably, a LysM RLK was firstly found involved in salt stress response. Moreover, key enzymes in the pathways of jasmonic acid biosynthesis, lipid metabolism and indole-3-acetic acid homeostasis were specifically affected by salt stress in salt tolerance genotype. These salt-responsive genes and biochemical pathways identified in this study could provide further information for understanding the mechanisms of salt tolerance in barley.

## 1. Introduction

Due to various biotic and abiotic stress factors under field conditions, crop plant yield reduction can reach more than 50% [1]. Among these abiotic stresses, salinity is the most severe environmental stress affecting more than 800 million hectares of land throughout the world [2, 3]. Unsuitable irrigation was the most significant reason leading to cultivated agricultural land salinization [4]. With the constantly growing world population, the demands for food are increasing rapidly, so it is an important global priority to improve the salt tolerance of crops [3]. The discovery of novel genes, the analysis of their expression patterns in response to salt stress, and the determination of their potential functions in salt stress adaptation will provide the basis of effective engineering strategies to enhance crop salt stress tolerance [5].

To cope with the detrimental effects of various abiotic stresses, crops have evolved many mechanisms to increase their tolerance, including physical adaptations, and interactive molecular and cellular changes [6]. The crops can switch on these mechanisms through a signal transduction pathway when they perceive environmental stress [7, 8]. Understanding the mechanisms of signal transduction is not only of fundamental importance to biology but also essential for the continued development of rational breeding and transgenic strategies to improve stress tolerance in crops [7]. 

During recent years, considerable attention has been directed toward elucidating the molecular basis of plant salt tolerance. Several important pathways involved in salt stress signal transduction have been identified from *Arabidopsis* and rice, such as the salt oversensitive (SOS) pathway [9, 10], the calcium-dependent protein kinase (CDPK) pathway [11], and the mitogen activated protein kinase (MAPK) pathway [12], the oxylipin pathway [13], and endoplasmic reticulum stress signaling [14]. Also, plant hormones, such as abscisic acid (ABA), ethylene, salicylic acid, and jasmonic acid, all play vital roles in salt-stress signaling and adaptation [15–17]. Considerable research has shown that different pathways are interconnected and coordinately regulate the plant response to biotic and abiotic stresses [6, 16, 18–20].

Barley (*Hordeum vulgare *L.) is an important crop usually used as human food, malt, and feed for animals [21] and is the most important field crop after rice, wheat and maize [22]. Among the cereal crops, barley is considered as a notably salt-tolerant cultivar [23, 24], which also shows considerable variation for tolerance towards salinity stress [3, 25]. Furthermore, the salt tolerance of barley varies with the plant growth stage. It is reported that the germination and young seedling stage is the most sensitive and affects the final yield. However, the barley exhibits an increased tolerance with age [24]. Investigating the salt-tolerance mechanisms in barley could facilitate a better understanding of the genetic basis of salt tolerance and therefore enable the effective use of genetic and genomic approaches to improve salt tolerance. 

Transcriptome analysis has always played a central role in elucidating the complexity of gene expression regulation. Among several transcriptome analysis methods, microarray technologies and RNA-seq have become the default popular methods of choices for genomewide transcriptome studies [26]. Although RNA-seq has recently become a preferred method of choice in whole transcriptome analysis, microarrays represent a well-established technology and have been widely used in the last decades and have provided a great deal of candidate genes for genetic engineering [24, 27, 28]. In the current study, a cDNA microarray technology was used to identify salt stress-regulated genes on a large scale and to clarify the complex molecular mechanisms of barley salt tolerance from two cultivated barley genotypes: Hua 30, a salt-sensitive cultivar, and Hua 11, a salt-tolerant cultivar. These cultivars were derived from microspores of progeny of the same parental cross and display highly similar phenotypes in developmental and responsive processes other than salt tolerance. Our previous work has disclosed that Hua 30 and Hua 11 showed evident difference in salt tolerance at distinct developmental stages [29–31]. Thus they provide excellent materials for barley salt tolerance research. According to gene expression patterns, the salt stress responsive genes can be broadly classified as early- and late-induced genes [8]. It is reported that the early responsive genes are important to environmental stress response [8, 32]. Previous studies have provided well understanding of transcription responses of barley to salinity stress after long time exposure, for example, 24 h [33, 34], 27 h [24], and 5 days [27]. However, few reports were available on the early responses [33]. To bridge the gaps, transcriptional analysis was performed at 6 h after salt treatment in two genotypes showing different tolerance to salinity.

## 2. Materials and Methods

### 2.1. Plant Cultivation and Salt Stress Treatment

Seeds of the salt-tolerant cultivar, Hua11, and the salt-sensitive cultivar, Hua30, were obtained from the Biotechnology Research Center of the Shanghai Academy of Agricultural Sciences. Uniformly sized seeds were selected, surface-sterilized in 75% ethanol for 1 min and rinsed several times, germinated in distilled water overnight, and then placed on moist filter paper of deep cell culture dishes in a growth chamber. After 7 d, seedlings at similar germination stage were suspended in a half-strength Hoagland solution with double iron (50 g·L^−1^). The pH of the solution was maintained within the range of 5–6.5 using KOH. Plants were grown in controlled conditions at 25/22°C day/night, 12 h photoperiod, and 70% humidity. The salt stress was imposed from the 11th day after germination. CaCl_2_ was added with NaCl to maintain an Na^+^/Ca^2+^ concentration ratio of 10 : 1 on a molar basis. In the preexperiment, 150, 200, 250, 300, and 400 mM NaCl gradient stress was applied on barley seedlings for three days to judge the optimum concentration for salt stress. According to the preexperiment results, 300 mM was chosen as our stress scale due to the more phenotypic changes observed between Hua 30 and Hua 11 than others. As mentioned before, on 11th day old seedlings were transferred either to half-strength Hoagland with 300 mM NaCl for salt stress treatment or to half-strength Hoagland solution without salt as the control. Roots and shoots from 15 plants were harvested separately from control and salt-stressed at 6 h after the treatment, frozen in liquid nitrogen, and then kept at −70°C for RNA extraction. 

### 2.2. Physiological Assays

According to the previous method, roots and shoots were harvested separately at 0 h, 6 h, 48 h, and 72 h after salt-stressed for the POD (peroxidase) and SOD (superoxide dismutase) activities determination.

The activity of POD was assayed by the method of Evans and Alldridge [35] with lesser modifications. The reaction mixture was treated in water bath at 37°C for 5 min and cooled in an ice bath. The total peroxidase activity was expressed as the increase in absorbance at 470 nm min^−1^ g^−1^ FW (0.01 OD = 1 enzyme unit).

The activity of SOD was assayed by the method of Droillard et al. [36] with measuring its ability to inhibit the reduction of nitroblue tetrazolium (NBT). The absorbance was measured at 560 nm of the reaction mixture. The volume of enzyme extract corresponding to 50% inhibition of the reaction was regarded as 1 enzyme unit.

### 2.3. RNA Preparations and Gene Chip Hybridization

Total RNA was extracted using the TRIzol reagent (Invitrogen, USA) according to the manufacturer's instructions. The quality of RNA was analyzed on a denaturing formaldehyde gel and confirmed by measuring the ratio of A260/A280 with the NanoDropND-1000 spectrophotometer.

Sample treatment, hybridization, and scanning of Affymetrix Barley 1 GeneChip with 22792 probe sets were carried out at the College of Life Sciences, Zhejiang University, as described in reference of Cheng et al. [37]. The SuperScript double-stranded cDNA synthesis kit (Invitrogen) was used to synthesize complementary DNA. A part of the double-stranded cDNA was used as a template to produce biotin-tagged cRNA by an Affymetrix GeneChip IVT labeling kit (Affymetrix). Fifteen micrograms of the biotin-tagged cRNA was separated into a size range of 35 to 200 bases following the manufacturer's instructions. Then 10 ug of this fragmented biotin-tagged cRNA was hybridized at 45°C with rotating for 16 hr to probe sets present on GeneChip Drosophila genome array. These genome arrays were washed and stained using streptavidin-phycoerythrin on an Affymetrix Fluidics Station 400, as described in the Affymetrix protocols, and then were scanned using the Hewlett-Packard GeneArray Scanner G3000. The hybridization data were analyzed using Microarray Suite version 5.0. Two biological replicates per treatment and per genotype were analyzed. 

### 2.4. Statistical Analysis of the Microarray Data

Raw intensity data were firstly normalized using the MAS5 algorithm, which allowed probe identifier present calls to be determined. Only those probe sets which called present in two replicates under treatment were included and then probe intensities were analyzed by the GC-RMA algorithm and log transform was carried out. Subsequently, the average log signal intensity values of two biological replicates for each sample were calculated and used for further analysis. Genes that had significant expression between the control and salt-treated roots or shoots of each variety were identified at *P* < 0.05 with an empirical Bayes' *t*-test using false discovery rate (FDR) for multiple testing corrections [38] and by using the empirical criterion of greater than a twofold change.

### 2.5. Probe Set Annotations, Gene Ontology Analysis, and Biochemical Pathways Analysis

The probe sets were annotated using HarvEST:Barley (version 1.77) assembly 21(HarvEST:Barley (http://harvest.ucr.edu/)), which can provide the best BLASTX hit against UniProt, TIGR rice, and TAIR *Arabidopsis* database with a cut-off threshold of E-20. The least number of probes matched selected for the annotation was 11. Blast2go (http://www.blast2go.com/b2glaunch) was used to analyze gene ontology and then KOBAS (KEGG-Ontology (KO-) Based Annotation System) was used to identify the most statistically significantly enriched KEGG pathways and the *Arabidopsis thaliana* genome was used as the background distribution.

### 2.6. Quantitative Real-Time PCR

The expression profiles of several important transcripts obtained from gene chip hybridizations were further validated by real-time PCR using the first strand cDNA synthesis from independently isolated RNA samples. A cDNA first strand was synthesized using M-MLV first strand kit (Invitrogen, cat no. C28025-032) following the manufacturer's instructions. Five micrograms of total RNA was converted into 40 *μ*L cDNA. Each cDNA was diluted 20 times and 2 *μ*L of cDNA was used for two-step PCR. PCR was performed with the SYBR Green Real-time PCR Master Mix (TOYOBO, code no. QPK 201) in a reaction volume of 20 *μ*L containing 2 *μ*L of diluted cDNA. Cycling was carried out on an Applied Biosystems StepOne Real-Time PCR System following the manufacturer's instructions. The ten genes randomly selected for validation only included those that had upregulated expression in response to salinity stress. Seven genes belonged to the shoot and the others belonged to the root. All PCRs were repeated three times for biological replicates at each sampling time point. A barley *actin* gene (forward primer: GCCGTGCTTTCCCTCTATG; reverse primer: GCTTCTCCTTGATG TCCCTTA) was used as a control for real-time PCR.

## 3. Results 

### 3.1. Response of Hua 30 and Hua 11 at Seedling Stage to Salinity Stress

**Figure 1:**
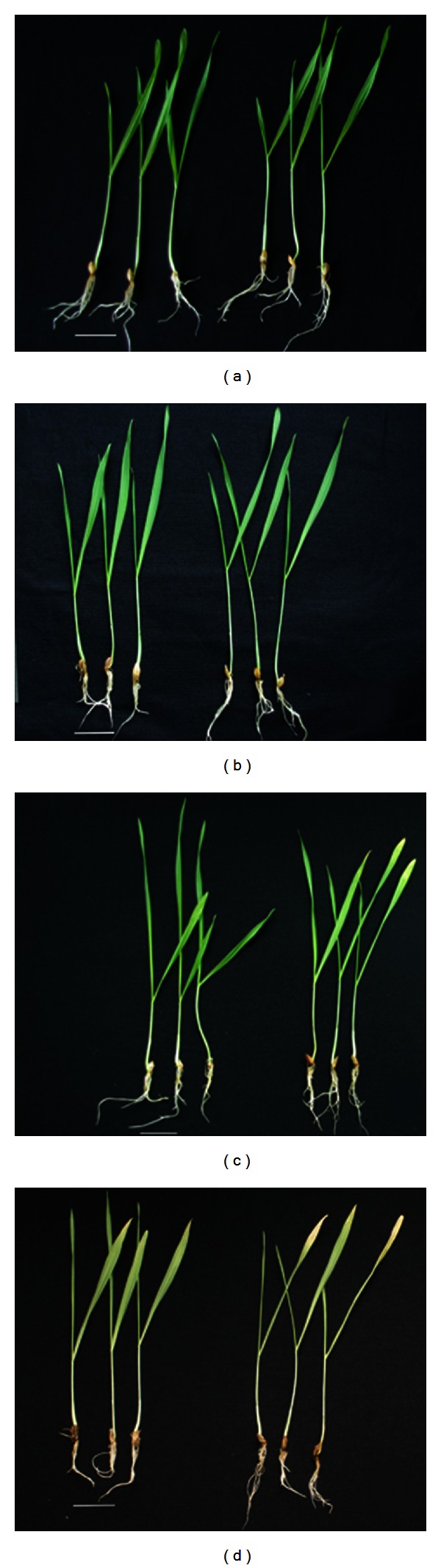
Barley phenotypic changes: (a) 12 d after germination without NaCl treatment. (b) After 6 h treatment with 300 mM NaCl, Hua 30 and Hua 11 have no phenotypic changes. (c) 10 d seedlings with 300 mM NaCl for 48 h. (d) 10 d seedlings with 300 mM NaCl for 72 h. Left: Hua 11, right: Hua 30, bar = 2.5 cm.

Salt tolerance in barley varieties of Hua 30 and Hua 11 was identified clearly at the microspore stage [29], germination stage [30], and seedling stage [31]. In this experiment, no significant difference at the seedling stage was observed between the two genotypes under normal conditions (Figure 1(a)), while under severe salt stress (300 Mm NaCl), phenotypic changes were observed in Hua 30 compared with Hua 11: first leaves apex turned yellow (Figure 1(c)), reduced growth (Figure 1(c)), and the severe dehydration of new leaves (Figure 1(d)).

### 3.2. Effect of Salt Stress on POD and SOD Activities in Barley

Salinity, like other environment stresses, also triggers plant to generate reactive oxygen species (ROS) [39]. However, stress-induced ROS accumulation is counteracted by enzymatic antioxidant systems or nonenzymatic low molecular metabolites [40]. Superoxide dismutase (SOD; EC 1.15.1.1) is a major scavenger of superoxide (O^2−^), and the products of its enzymatic action are H_2_O_2_ and O_2_. Subsequently the hydrogen peroxide produced is scavenged by a variety of peroxidases (POD: EC 1.11.1.7). Therefore, the activities of SOD and POD play important roles in the protection of barley plants from salt stress

**Figure 2:**
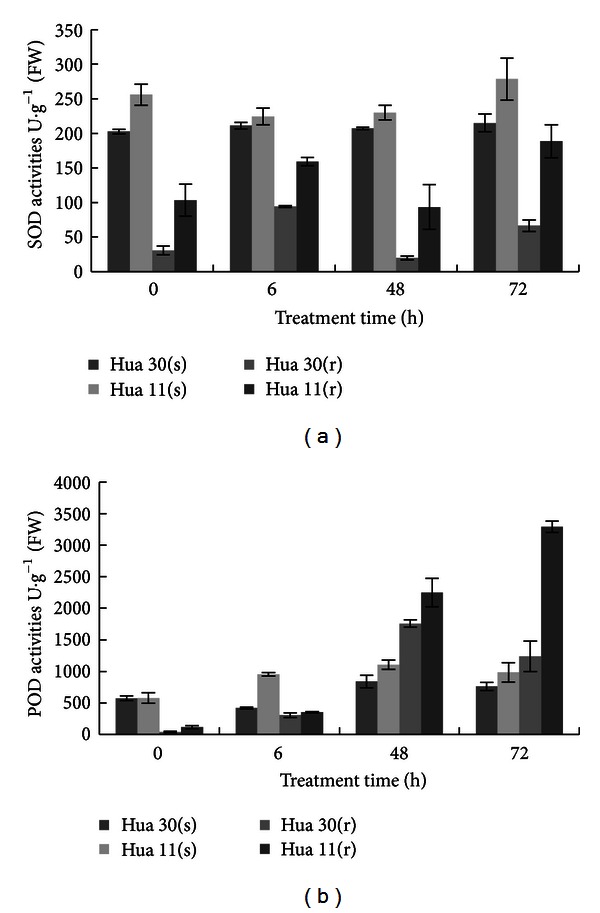
Changes in SOD (a) and POD (b) activities in shoots and roots from Hua 30 and Hua 11. Vertical bars represent standard error of means. s: shoot; r: root.

Before treatment, the background level of shoot SOD activity was high in Hua 11 than that in Hua 30 (Figure 2(a)). Salt treatment had no significant impact on SOD activity in the shoot tissue of Hua 30, whereas SOD activity in shoot tissue of Hua 11 was decreased slightly and then increased obviously. Similarly, the background level of root SOD activity was high in Hua 11 than that in Hua 30 before salt treatment. During 72 h salt treatment, similar fluctuation in SOD activity was observed in Hua 11 and Hua 30 roots, but the SOD activity always was much higher in Hua 11 root than in Hua 30 root. 

Before salt treatment, the POD activity was a little higher in root but nearly similar in shoot of Hua11 as compared with that in Hua 30 corresponding tissues (Figure 2(b)). It rose steadily after salt treatment in both shoot and root tissues of Hua 11 while it was fluctuated in both tissues of Hua 30. It was particularly higher in Hua 11 root than in Hua 30 root after 72 h salt.

In conclusion, the SOD and POD activities in Hua 11 were higher than Hua 30 in both shoots and roots, which provides sound biochemical basis for the higher tolerance of Hua 11 to salt stress than Hua 30.

### 3.3. Expression Profiles of Salt-Responsive Genes in Barley

**Figure 3:**
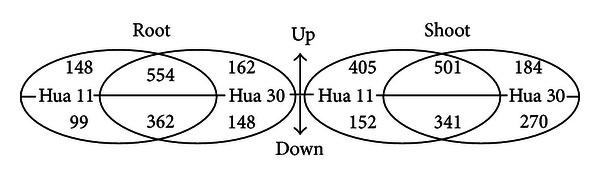
Number of differentially expressed probe sets in different barley genotypes tested under salt stress. The Venn diagrams shows the number of probe sets up or down in shoot or root tissues of Hua 11 and Hua 30 in response to salt stress at level of 2fold or more and a *P* value <0.05. The redundancies probe sets are included only once in this Venn diagram.

Changes of gene expression in Hua 11 and Hua30 under salt stress were investigated using Barley 1 microarrays. A total of 1853 and 1473 differentially regulated probe sets were found in the shoot tissue and root tissue, respectively, of the two genotypes after 6 h of salt treatment (Figure 3). In root tissue, the number of differentially regulated probe sets in Hua 11 and Hua 30 was 1163 and 1226. By contrast, 916 coregulated probe sets being identified in two genotypes, with only 247 probe sets were identified in Hua 11 and 310 probe sets in Hua 30. In the shoot tissue, the number of salt responsive probe sets in Hua 11 and Hua 30 was 1399 and 1296. By comparison, 842 coregulated probe sets being found in two genotypes, with only 557 probe sets were found in Hua 11 and 454 probe sets in Hua 30 (Table 1).

These significantly changed probe sets related to signal transduction, transcription factors, and compatible and secondary metabolites were classified (Table 1). The number of differentially regulated probe sets related to signal transduction was 176 and 103 in shoot and root tissues, respectively, which only in Hua 11 was 59 and 19 while only in Hua 30 was 38 and 21. For the salt responsive transcription factors, 91 and 49 probe sets were found in shoot and root tissue, which only in Hua 11 was 30 and 6 while only in Hua 30 was 16 and 10. Regard to the compatible and secondary metabolites, there were 32 and 33 different expression probe sets were found in shoot and root tissue, which only in Hua 11 was 25 and 11 while only in Hua 30 was 13 and 10.

#### 3.3.1. Signal Transduction Elements Responsive to Salt Stress in the Salt-Tolerant Genotype

It was suggested that genes that showed a significant response to salt in the salt-tolerant genotype Hua 11, but did not respond (un-altered or even reversely altered) in the salt-sensitive genotype Hua 30 are likely to be related to salt tolerance [41]. To investigate the signal transduction process in barley under salt stress conditions, differentially-expressed signaling-related genes were subjected to analysis. 59 genes in shoot tissue and 18 genes in root tissue involved in signaling pathways were identified in the salt-tolerant genotype Hua 11 (Table 2). These genes were classified into four major groups including kinases, phosphatases, hormone-related genes and others.

The first group is kinases, and contained receptor-like kinases (RLKs), Ser/Thr kinases, CIPK, CDPK, MAPK and others kinases, with 14 RLKs identified as salt stress-responsive genes in the Hua 11 shoot tissue and 0 in the root tissue. All of these RLKs were upregulated by salt treatment. 10 Ser/Thr kinases (9 up and 1 down) in shoot tissue and 4 (3 up and 1 down) in root tissue were identified as salt-responsive genes in Hua 11. In addition, two CDPKs (calcium-dependent protein kinase) and one MAPK (mitogen-activated protein kinase) were identified in shoot tissue, and one CIPK was identified in root tissue. The remaining kinases include two ribose-phosphate pyrophosphokinases (Contig5081_at and Contig8025_at), one amino acid kinase (Contig12389_s_at), one phosphatidylinositol-4-phosphate 5-kinase (Contig19429_at), one phosphatidylinositol kinase and FAT-containing domain protein (Contig8975_at), one protein kinase domain-containing protein (Contig11835_at), one unknown function DUF1296 domain-containing protein (Contig5785_at), one tyrosine protein kinase domain-containing protein (Contig19683_at) in shoots, and one phosphomethylpyrimidine kinase (Contig15880_at) in root tissue. These genes were upregulated in Hua 11.

The second group is phosphatases. They were divided into two major classes: protein serine/threonine phosphatases and protein tyrosine phosphatases. 7 serine/threonine phosphatases were identified in shoot tissue (6 up and 1 down) and one up-regulated gene was identified in root tissue. Only one protein tyrosine phosphatase (Contig9830_s_at) was identified in shoot tissue.

The third group is hormone-related genes, which included jasmonic acid (JA), ethylene, gibberellins, auxin, and cytokinin. Most of these genes were up-regulated.

The remaining signal-related genes were second messengers and response regulators. Only one of the 7 remaining signal-related genes was downregulated in the shoot tissue, and one gene out of 2 was downregulated in the root tissue.

#### 3.3.2. Transcriptional Regulation Responsive to Salt in the Salt-Tolerant Genotype

To understand the transcriptional regulation in salt stress response in the salt-tolerant genotype Hua 11, 30 genes identified in the shoot and 6 genes identified in the root of Hua 11, all of which encoding transcription factors, were further characterized (Table 3). These results suggest the existence of many transcriptional regulatory mechanisms in high-salinity stress signal transduction pathways. Among these salt stress-inducible and Hua 11-specific transcription factors in shoot tissue, there are two ZIM motif family genes, nine Zinc finger family genes, four WRKY family genes, three MYB family genes, two basic helix-loop-helix (bHLH) family genes, two C-repeat binding factors, two NAC family genes, two basic leucine zipper (bZIP) family genes, three AP2 family genes, and one whirly family gene. Most of these were up-regulated, with the exception of four genes. Six salt responsive genes coding transcription factors were found Hua 11-specific in root tissue, including two Zinc finger family genes, one WRKY family gene, one MYB family gene, one bZIP family gene, and one Homeobox-leucine zipper gene. Only two genes (one MYB family gene, one bZIP family gene) were down-regulated in root.

#### 3.3.3. Metabolism Responsive to Salt in the Salt-Tolerant Genotype

The major roles of compatible solutes and plant secondary metabolites are to protect plants in order to survive biotic and abiotic stresses. In order to assess the compatible solutes and secondary metabolites in salt stress response in the salt-tolerant genotypes, 15 genes in shoots and 11 genes in roots were identified in the tolerant genotype Hua11 (Table 4). The compatible solutes are all up-regulated in shoots and roots. One proline-related gene and 4 sugar metabolite-related genes were found in shoot tissue, and one sugar metabolite related gene was found in root tissue. The secondary metabolite genes have a simple classification that includes three main groups. The first group is phendic: one flavonoid-related gene (Contig11944_at), two simple phenolic genes (Contig24054_at; Contig24228_at), and one quinine-related gene (Contig12883_at) were identified in the shoot tissue; three flavonoid-related genes (Contig25479_at; Contig17030_at; Contig22018_at) and three simple phenolic-related genes (Contig8345_at; Contig3308_at; Contig6733_at) were identified in the root tissue. The second group is terpenoid: only two genes (HT09C21r_s_at; Contig3183_at) were identified from shoot tissue. The third group is nitrogen-containing compounds: three alkaloids-related genes (Contig13038_at; Contig13228_at; Contig14427_at) in shoot tissue and two genes (Contig2900_at; Contig23347_at) in root tissue were identified. Three amine-related genes (Contig5994_s_at; Contig13254_at; Contig6067_at) were also identified from root tissue.

#### 3.3.4. Gene Ontology Analysis of the Salt Stress Responsive Genes

**Figure 4:**
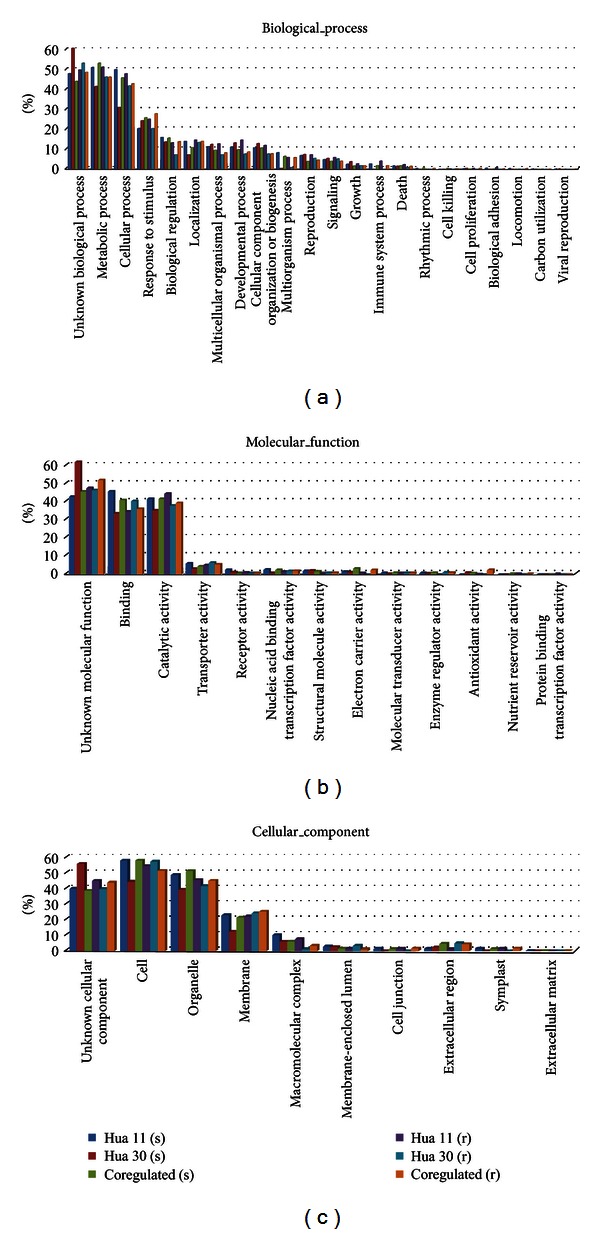
Gene Ontology (GO) classification of the differentially expressed genes. Gene Ontology (GO) assignment to these genes based on high-score BLASTX matches to the *Arabidopsis* proteins (TAIR) is classified into three main GO categories (biological process, molecular function, and cellular component); the left *y*-axis indicates the percentage of a specific category of genes in that main category (GO level** **= 2). s: shoot, r: root.

To investigate the identity of the differentially expressed genes, these genes were annotated by the HarvEST software version 1.77 based on the best BLAST matches to UniProt, *Arabidopsis*, and rice gene sequence. As a result, most of the differentially expressed genes could be assigned to a best scoring BLAST hit and more than 70% genes had the GO assignments. Using blast2go software, these differentially expressed genes with the GO assignments were classified into different GO groups including molecular function, biological process, and cellular component (Table 5 and Figure 4). 

Regarding biological processes, genes involved in cellular process, metabolic process, cellular process, and response to stimulus were highly represented. The well-represented molecular functions were binding and catalytic activity. For cellular component, the most represented categories were cells, organelles, and membrane (Figure 4). 

#### 3.3.5. Biochemical Pathways Affected by Salt Stress in the Salt-Tolerant Genotype

A lot of differentially expressed genes were isolated in our study. In order to assess the functional roles of these salt-responsive genes involved in biochemical pathways, specific biochemical metabolisms affected by salt stress only in the salt-tolerant genotype were identified by KO-Based Annotation System (KOBAS), which can provide the most frequent and statistically significantly enriched pathways. In the shoot tissue of Hua 11, 13 pathways were identified from the up-regulated genes with the *P* value <0.05, including 38 genes. Meanwhile, 6 pathways were identified in the root tissues with the *P* value <0.05; 7 genes were involved (Table 6). These pathways came from Kyoto Encyclopedia of Genes and Genomes (KEGG) pathway databases and BioCyc databases. In the shoot tissue, the pathways about PRPP biosynthesis I, jasmonic acid biosynthesis, 13-LOX and 13-HPL pathway, very long chain fatty acid biosynthesis and sucrose biosynthesis were classified into biosynthesis in BioCyc databases. All others came from KEGG pathway databases which can be classified into three categories. The first one was metabolism containing N-glycan biosynthesis, linoleic acid metabolism, and sphingolipid metabolism. The second category was genetic information processing and the protein processing in endoplasmic reticulum, ribosome biogenesis in eukaryotes, RNA transport, and spliceosome which were included. The plant-pathogen interaction belonging to organismal systems was the last category. In the root tissue, all the pathways came from the BioCyc databases except the lipid metabolism which was derived from KEGG pathway databases and belonged to the category of metabolism.

### 3.4. Validations of Microarray Results by Real-Time PCR

**Figure 5:**
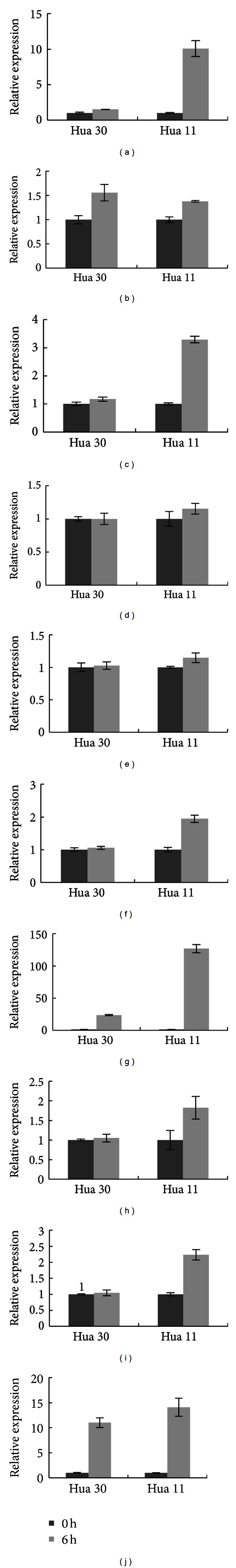
Verification of microarray results by real-time PCR. Real-time PCR analysis of ten selected genes. Actin was used as the internal control. 0 h: control plants. 6 h: salt stress plants. (a) Contig5422_at; (b) Contig5740_at; (c) Contig6159_at; (d) Contig7044_at; (e) Contig10690_at; (f) Contig20719_at; (g) HB03A08_T3_at; (h) Contig13791_at; (i) Contig24300_at; (j) HC02E04_T3_at.

To confirm the microarray results, real-time PCR was conducted on 10 randomly selected salt-responsive genes based on the microarray (Table 7). Seven genes belonged to those differentially expressed in shoot and three genes belonged to those in root. The real-time PCR analysis showed that only result of one gene (Contig5740_at) did not agree with the microarray data, which indicated that there was a good consistency between the real-time PCR and microarray results (Figure 5).

## 4. Discussion

Using the transcriptional profiling of Barley1 chip, it found that the salt tolerant barley: Hua 11 and salt sensitive barley: Hua 30 exhibited a diverse transcriptional response under the salt stress. Interestingly, comparison of the expression levels of gene between Hua 11 and Hua 30 revealed that 916 (62%) genes and 842 (45%) genes were co-regulated, respectively in root and shoot tissue. These genes might be responsible for barley intrinsic tolerance to salt stress. The most attention is that the number of salt responsive genes identified from shoot tissues (1853) more than from root tissues (1475). The similar results had got in rice [41, 42], which demonstrates the tissue-specific nature of the salinity response not only in rice but also in barley. Mangelsen et al. [43] reported that the early heat stress respond in barley was divide into three distinct temporal phases termed sensing and signal transduction, primary heat stress response, and heat stress adaptation. Although the salt stress is differing from the heat stress, the similar mechanism will be shared among the abiotic stresses. A reasonable explain for this phenomenon may be the salt responsive gene from root mostly in the phase of adaptation while the genes identified from shoot were involved in the first two phases after 6 h of salt stress. The salt-responsive genes only expressed in the salt tolerant genotype might participate in the special process of salt tolerance in barley. Comparison of gene expression profiles between the salt tolerant genotype and salt sensitive genotype under salt stress is essential for the elucidation of the salt response networks in barley.

### 4.1. Signal Transduction and Hormonal Regulation under Salt Stress in Barley

Stress signal transduction plays a crucial role in plant stress response [32]. Kinases and phosphates can turn on or off salt stress responses by reversible phosphorylation to activate transcription factors and other functional genes [32, 44]. In this study, a large number of signaling-related elements were identified.

Receptor-like kinases (RLKs) play vital roles in plant development, hormone perception, and biotic or abiotic stress responses, which belong to the group of earliest response genes in plants under stress conditions [45]. The architecture of receptor-like protein kinases (RLKs) indicates that RLKs can perceive and transmit external signals from the environment and then activate downstream signaling cascades to induce appropriate cellular responses [46, 47]. The RLKs have been shown to be highly induced by salt in *Arabidopsis* and rice [45, 48]. Among these RLKs, a somatic embryogenesis receptor kinase 1 (SERK 1) up-regulated in shoot tissue of Hua 11 was involved in the brassinosteroid (BR) signaling pathway, which led to a broader range of functions in plant responses to biotic and abiotic stimuli [49, 50]. A WAK receptor-like cytoplasmic kinase, as a signaling linker between the cell wall and the cytoplasm, was involved in biotic and abiotic stress responses [51, 52]. Interestingly, a WAK-like kinase in Arabidopsis, WAKL4, has been reported that it was significantly up-regulated by salt stress. This result was similar to our microarray data, Contig12629_s_at, a WAK receptor-like cytoplasmic kinase also up-regulated after salt stress [53]. The LysM receptor-like kinase (LysM RLK) performs a critical function in chitin signaling and fungal resistance [46, 54]. However, an LysM RLK was remarkably induced by salt stress in this study and it was firstly found involved in salt stress response. This result may indicate that the LysM receptor-like kinase plays key roles in the crosstalk between biotic and abiotic stress signaling.

MAPKs involved in MAPK pathways are activated by diverse abiotic stresses and act as a link between upstream receptors and downstream targets [55, 56]. Ca^2+^ is implicated as a “second messenger” in signaling in responses to both biotic and abiotic stress [57–59]. Calcium-dependent protein kinases (CDPKs) can bind and sense calcium ions directly to induce the downstream signaling in response to different stresses [11]. The CBL-CIPK signaling system, a newly emerging plant-specific and Ca^2+^-dependent network, mediates abiotic stress tolerance, for substances such as salt [60]. In this study, MAPK and CDPK signaling pathways were up-regulated under salt stress, which were similar to the result of Zhang et al. [32]. Calcium sensors and their interactive proteins were also identified in salt-tolerant genotype of Hua 11. These results indicated that MAPK pathway and Ca^2+^-dependent signaling pathway might play important roles in early response of barley under severe salt stress.

Protein phosphatases, by opposing the functions of the protein kinases, also have a general mechanism for transmitting signals from the extracellular surroundings to the interior of the cell [64]. PP2B phosphatase calcineurin (CaN), a key component of Ca^2+^-dependent signal transduction pathway, functions to restrict intracellular Na^+^ accumulation to mediate salt adaptation in plants [65]. PP2Cs not only act as negative regulators involved in the ABA signal transduction pathway, but also function as regulators of mitogen-activated protein kinase pathway and receptor kinase signaling [66], while these pathways all play important roles in stress signaling. The protein tyrosine phosphatases were another type of phosphatases which also are involved in the mitogen-activated protein kinases pathway [67]. It has been reported that a tyrosine-specific protein phosphatase (*AtPTP1*) is positively regulated by high-salt exposure in Arabidopsis, which was similar to our result [67]. In a word, all of these suggested that protein phosphatases may have crucial roles in NaCl stress signaling.

Phytohormones, such as abscisic acid (ABA), jasmonic acid (JA), and ethylene (ET), regulate the protective responses of plants against abiotic stresses by synergistic and antagonistic actions [16]. In this study, a set of salt-regulated hormone-related proteins were identified both in shoot and root tissues in Hua 11. Among them, three lipoxygenases were up-regulated in shoot tissue. The lipoxygenases initiated the plant oxylipin pathway and then produced jasmonic acid (JA), which participates in the defense responses against biotic and abiotic stresses [68–70]. Ethylene (ET) was also found to be involved in many abiotic stress responses [71]. In this study, one cytokinin dehydrogenase was significantly up-regulated in the root tissue of Hua 11 by NaCl-treatment. Due to the fact that the concentrations of endogenous cytokine can be reduced by the cytokinin dehydrogenase, the antioxidative enzyme activity increases as cytokine decreases [72]. Therefore, the barley salt stress tolerance may be improved by the enhancement of antioxidant enzymes. The result indicated that there is a potential link between salt stress and cytokinin signaling in barley.

According to the aforementioned, the salt stress signal transduction was very complex. These various signal molecules can act alone or together to improve the salt tolerance of barley. 

### 4.2. Transcription Regulations under Salt Stress in Barley

Stress-related gene induction occurs principally at the transcriptional level, and regulation of the temporal and spatial expression patterns of specific stress-related genes is an important part of the plant stress response [73, 74]. In this research, a number of differentially expressed genes encoded transcriptional factors that were distinguished in the salt tolerant genotype of Hua 11. Among them, the ZIM motif family of genes, which is now known as TIFY family, was found to be responsive to one or more abiotic stresses [75]. WRKY transcription factors have been confirmed to be involved in various abiotic stress responses [73, 76, 77]. The AP2/EREBP family of transcription factors was shown to mediate distinct responses to abiotic stresses by hormone-dependent gene expression [78]. The bHLH (Basic Helix-Loop-Helix) proteins were found to play an important role in the ABA-mediated signal transduction pathway and in secondary metabolism [79], which also regulates the adaptive response of plants to abiotic stresses [80]. In addition, NAC transcription factors have been shown to function in relation not only to plant development but also to abiotic and/or biotic stress responses [81]. Whirly transcription factors are an important component of the SA (salicylic acid) signaling pathway-regulated defense of gene expression [82]. The CBF (C-repeat binding factor) transcription factors, also known as DREB1s, were involved in responses to freezing, drought, and high-salt stress tolerance [83, 84]. The HD-Zip genes were potentially involved in both ABA-dependent and -independent drought-responsive signaling pathways [85]. 

Among the previously mentioned transcription factors, one C-repeat binding factor and one bHLH transcription factor showed a special pattern compared to other transcription factors. They were both up-regulated in the shoot tissue of Hua 11 and down-regulated in the shoot tissue of Hua 30 after 6 h of salt stress. They are, therefore, more likely to be related to salt tolerance than genes that are responsive only in Hua11 and not in Hua 30. The potential roles of these two transcription factors in salt tolerance should be further investigated. All of these transcription factors identified from the salt-tolerant genotype of Hua 11 showed complicated transcriptional regulatory networks of salt responses in barley, as they have been shown to perform crosstalk with phytohormones and secondary metabolism.

### 4.3. Compatible Solutes and Secondary Metabolites under Salt Stress in Barley

Under salt stress, crops can adjust their osmotic pressure by synthesizing compatible organic solutes to ease the damage brought upon by salt [62]. Proline is one of the most widely studied compatible solutes, and its accumulation was observed in various organisms under conditions of salt stress [62, 86]. However, in this study, only one proline-related gene (Contig2524_s_at) was found in the shoot tissue of the salt-tolerant genotype Hua 11, which encoded a proline degradation enzyme. A reasonable explanation for this has not been found at present. Sucrose and fructose also have the ability to balance the osmotic potential of Na^+^ and Cl^−^ [87].

The secondary metabolites have a protective role against biotic and abiotic environmental stresses [63]. The flavonoid biosynthesis pathway was identified in rice salt-sensitive genotypes but not in salt-tolerant genotypes [42]. However, in this study, the flavonoid biosynthesis pathway was induced not only in the salt-tolerant Hua 11 but also in the salt-sensitive Hua 30. The exact roles of flavonoids during barley salt stress are therefore not certain at present and need further study.

### 4.4. Specifically Affected Biochemical Pathways in Salt Tolerant Genotype

#### 4.4.1. The Function of the Genetic Information Processing

Gene regulation is an essential process in the development and maintenance of a healthy body. The regulation of gene expression allows a cell to express specific proteins when needed to adapt to trigger developmental pathways, respond to environmental stimuli, and so on. In this study, four pathways involved in genetic information processing were significantly identified from the salt tolerant genotype, which means that the salt tolerant genotype could make an active response to salt stress. Meanwhile, the more differentially expressed genes were identified in salt tolerant genotype than sensitive genotype which also provides an evidence to support these results.

#### 4.4.2. Jasmonic Acid (JA) Pathway Was Induced in Shoot of the Salt Tolerant Genotype

Jasmonic acid (JA) and its cyclic precursors and derivatives are collectively referred to as jasmonates (JAs). JAs are lipid-derived compounds with signal functions that mediate plant stress responses and development processes [88]. Linoleic acid plays an important role as a precursor to the signal molecule of jasmonate [89]. 13-LOX and 13-HPL pathway can produce Jasmonic acid [90]. In our study, three genes were involved in Jasmonic acid biosynthesis pathway and have outstanding respone to salt stress in tolerant barley (Table 6). A similar observation has also been made in rice. Kang et al. [91] found that the concentration of endogenous JA in salt-tolerant rice was higher than salt-sensitive one at the various salt stress. Walia et al. [24] reported that salt stress can induce JA pathway-related genes in barley shoot tissue and the up-regulated lipoxygenase gene was also included. According to our result, identification of JA pathway is a prominent characteristic of the salt tolerant barley of Hua 11.

#### 4.4.3. Lipid Metabolism and Salt Stress

Sphingolipids are not only a crucial part of the membrane lipids but also serve indispensable roles as signaling molecules that control several cellular processes, for example, stress responses [92]. Very long chain fatty acids (VLCFA), which are essential precursors for sphingolipids, confer vital function in sphingolipid signaling [92]. In our study, the sphingolipid metabolism and very long chain fatty acid biosynthesis were identified in shoot tissue. It is worth noting that among the four enzymes, 3-ketoacyl-CoA synthase (KCS) was the first rate-limiting step in the biosynthesis of very long chain fatty acids (VLCFA). Joubès et al. [93] found that 3-ketoacyl-CoA synthase 6 gene had an increased expression in response to the salt stress in *Arabidopsis thaliana*. 

Phospholipids constitute the major part of cell membrane which is the first barrier that isolates cells from their environment and are a primary target for damage during abiotic stress [94]. In response to reducing the abiotic stress damage, the organisms can increase the level of their unsaturated fatty acids to keep the appropriate fluidity of cell membrane by converting saturated fatty acids [95]. It was reported that salinity stress could induce the high level expression of desaturase in yeast, which was consistent with as increased unsaturated fatty acids in the cell membranes [96]. Overexpression of two omega-3 fatty acid desaturases (either *FAD3* or *FAD8*) in tobacco could increase the various abiotic stresses including salt stress [97]. Moreover the high-salt treatment also induces the accumulation of two omega-3 fatty acid desaturase genes transcripts (*ZmFAD7* and *ZmFAD8*) in maize roots [98]. In this study, one omega-3 fatty acid desaturase was up-regulated in root of tolerant genotype, which possibly increased the content of unsaturated fatty acids to maintain the fluidity of cell membrane under the salt stress.

#### 4.4.4. Indole-3-Acetic Acid Homeostasis, and Salt Stress in Root Tissue

Indole-3-acetic acid (IAA) is an auxin that plays an important role in cell wall expansion, growth/development and responses to environmental stresses [99]. In vascular plants, most IAA exists in a conjugated form that serves as a storage form of auxin [100]. In present study, an IAA-amido synthetase that maintains auxin homeostasis by conjugating excess IAA to amino acids was identified from the root of salt tolerant genotype. The same result was also found in salt resistant maize, which can maintain IAA concentration in roots under the salt stress [99]. The IAA homeostasis adaptation may provide a relatively favorable condition for the salt tolerance variety growth.

## 5. Conclusion

Using the transcriptional profiling of Barley1 chip, it was found that the salt tolerant barley: Hua 11 and salt sensitive barley: Hua 30 exhibited diverse transcriptional responses under the salt stress. In our research a lot of salt stress-related genes were induced by other abiotic stresses, supporting current view of cross talk between several kinds of abiotic stresses. Plants have evolved sophisticated mechanisms to respond positively to the various abiotic and biotic stress, thus the salt-responsive genes only up-regulated in the salt tolerant genotype might participate in the specific processes of salt tolerance in barley. These genes could serve as “candidate genes” not only for further comprehending of molecular mechanisms of salt stress tolerance but also for improving the salt tolerance of barely or other crops by gene manipulation. Pathway analysis unraveled that lipid metabolism plays an important role in shoot and root tissue of salt tolerant genotype to resistant to salt stress. Jasmonic acid pathway induced by salt stress had been found in previous study [24], which also supported by our result. Maybe it has a vital role for the salt tolerant genotype, which will be as an interesting point for the future research.

Besides, about 25% salt responsive genes identified from our experiment (24% in shoot and 28% in root) were homologous to function-unknown genes or without homologs, which probably play important roles in barley under the salt stress. These data will serve as a valuable resource for further salt stress study by methods of molecular genetics, such as RNA interference or the gene overexpression.

## Figures and Tables

**Table 1:** The classification of the significantly changed probe sets related to signal transduction, transcription factors, and compatible and secondary metabolites.

Class description	No. of probes	Reverse expression of probes	No. of signal transductions	No. of transcription factors	No. of compatible and secondary metabolites
Upregulation in shoot	1090				
In Hua 11	906		52 + 52	26 + 35	13 + 22
In Hua 30	685	2	11 + 52	6 + 35	10 + 22
Downregulation in shoot	763				
In Hua 11	493		7 + 27	4 + 10	2 + 10
In Hua 30	611		27 + 27	10 + 10	3 + 10

Upregulation in root	864				
In Hua 11	702		14 + 38	4 + 19	7 + 21
In Hua30	716	0	14 + 38	7 + 19	4 + 21
Downregulation in root	609				
In Hua 11	461		5 + 25	2 + 14	4 + 12
In Hua 30	510		7 + 25	3 + 14	6 + 12

The number of probe sets up or down in shoot or root tissues of Hua 11 and Hua 30 in response to salt stress at level of 2-fold or more and a *P* value <0.05 was shown in this list. The annotation of probe sets was obtained by the best BLASTX hit against UniProt, TIGR rice, and TAIR Arabidopsis database. Functional classifications are defined according to Jiang and Deyholos [61], Rai et al. [62], and Mazid et al. [63]. The number in front of + was represented as the no. of probe sets only regulated in Hua 11 or Hua 30; the number behind + was represented as the no. of probe sets regulated both at Hua 11 and Hua 30. The redundancies probe sets are included only once in this list.

**Table 2:** Signal transduction responsive to salt stress in tolerant genotype.

Signal transduction components	Probe sets differentially expressed in shoot	Probe sets differentially expressed in root
Kinase		
Receptor-like kinase	Contig10249_at; Contig12629_s_at; Contig14350_at; Contig20719_at; Contig20799_at; Contig21807_at; Contig5000_x_at; Contig5422_at; Contig9077_at; Contig9408_at; HT05J08u_at; HT08K09u_at; Contig17366_at; rbaal11f18_at ↑	
Ser/Thr kinase	bags23d05_s_at; Contig13525_at; Contig4996_at; Contig13917_at; Contig4999_at; Contig13890_at; Contig25378_at; Contig7487_at; Contig8790_at ↑	Contig22232_at; HS16D10u_at; Contig13746_at ↑
	Contig9756_at ↓	Contig7535_at ↓
CIPK		Contig14822_at ↑
CDPK	Contig15719_at; Contig8546_s_at ↑	
MAPK	Contig14854_at ↑	
Others	Contig11835_at; Contig12389_s_at; Contig19429_at; Contig5081_at; Contig5785_at; Contig8025_at; Contig8975_at; Contig19683_at ↑	Contig15880_at ↑
	Contig23440_at ↓	Contig15771_at; HU08A18u_at ↓

Phosphatase		
Ser/Thr protein phosphatase	Contig4606_at(PP1); Contig10510_at(PP2B); Contig10850_at; Contig12379_at; Contig9265_at(PP2C); HVSMEk0009P03r2_at ↑	Contig18869_at ↑
	Contig4275_at (PP2B) ↓	
Protein tyrosine phosphatase	Contig9830_s_at ↑	

Hormone related		
JA	Contig22790_at; Contig1737_at; Contig2305_at; Contig2306_s_at ↑	
Ethylene	Contig13422_at ↓	Contig10113_at ↓
GA	Contig5444_s_at ↑	Contig7067_at; HVSMEb0001K06r2_s_at ↑
Auxin		Contig12102_at; Contig24931_at; Contig6407_s_at ↑
	Contig15125_at; Contig2503_s_at ↓	
Cytokinin		Contig24300_at ↑

Others		
Second messengers	AJ250283_at; Contig24334_at; Contig6611_at; Contig7549_at; Contig9645_at; Contig15452_at ↑	Contig12137_s_at ↑
	Contig18984_s_at ↓	Contig8468_at ↓
Response regulator		Contig11872_at ↑

↑ upregulation; ↓ downregulation. The probe sets showed in this list were identified at level of 2-fold or more and a *P* value <0.05.

**Table 3:** Transcription factors responsive to salt stress in tolerant genotype.

Classification of transcription factors	Probe sets differentially expressed in shoot	Probe sets differentially expressed in root
ZIM	Contig11225_at; Contig4813_at; ↑	
Zinc finger	Contig10187_at; Contig11855_at; Contig14085_at; Contig17951_at; Contig18088_at; Contig6585_at; Contig16225_at ↑	Contig12554_at; Contig20021_at ↑
	Contig20287_at; HZ51F23r_at ↓	
WRKY	Contig13375_at; Contig23697_at; Contig4386_at; HB25K10r_at ↑	Contig13268_at ↑
MYB	Contig3326_s_at; Contig9124_at ↑	
	HM12H05r_at ↓	Contig14220_at ↓
bHLH	Contig7031_at; Contig6159_at ↑	
CBF	Contig2479_at; Contig19472_at ↑	
NAC	Contig10340_at; Contig5740_at ↑	
Bzip	Contig10690_at ↑	
	Contig9253_at ↓	Contig11402_s_at ↓
AP2	Contig2471_at; Contig2470_s_at; Contig7722_at ↑	
Whirly	Contig8769_at ↑	
HD-zip		Contig13590_at ↑

↑ upregulation; ↓ downregulation. The probe sets showed in this list were identified at level of 2-fold or more and a *P* value <0.05.

**Table 4:** Compatible solutes and secondary metabolites responsive to salt stress in the salt tolerant genotype.

Classification of compatible solutes and secondary metabolites	Probe sets differentially expressed in shoot	Probe sets differentially expressed in root
Proline	Contig2524_s_at ↑	
Sugar metabolite	Contig460_s_at; Contig6623_s_at; Contig9965_at Contig12208_at ↑	Contig11241_at ↑
Phendic	Contig24054_at; Contig12883_at; Contig24228_at; ↑	Contig25479_at; Contig17030_at; Contig3308_at; Contig8345_at ↑
	Contig11944_at ↓	Contig22018_at; Contig6733_at ↓
Terpenoid	HT09C21r_s_at ↑	
	Contig3183_at ↓	
Nitrogen containing compounds alkaloids and amines	Contig13038_at; Contig13228_at; Contig14427_at; Contig6067_at ↑	Contig2900_at; Contig5994_s_at ↑
		Contig23347_at; Contig13254_at ↓

↑ upregulation; ↓ downregulation. The probe sets showed in this list were identified at level of 2-fold or more and a *P* value <0.05.

**Table 5:** Gene Ontology (GO) classification of the salt responsive genes.

	Total differentially expressed genes	The no. of genes with GO terms	The no. of genes involved in biological process	The no. of genes involved in molecular function	The no. of genes involved in cellular component
Hua 11 (s)	557	416 (74.7%)	320 (57.5%)	340 (61.0%)	335 (60.1%)
Hua 30 (s)	454	343 (75.6%)	210 (46.3%)	198 (43.6%)	201 (44.3%)
Coregulated (s)	842	656 (77.9%)	513 (60.9%)	492 (58.4%)	517 (61.4%)
Hua 11 (r)	247	174 (70.4%)	138 (55.9%)	140 (56.7%)	136 (55.1%)
Hua 30 (r)	310	231 (74.5%)	164 (52.9%)	179 (57.7%)	187 (60.3%)
Coregulated (r)	916	654 (71.4%)	521 (56.9%)	483 (52.7%)	515 (56.2%)

s: shoot; r: root.

**Table 6:** KOBAS-based pathway analysis of the up-regulated genes affected by salt stress in the tolerant genotype.

The pathway and the gene accession number	Gene description	*P* value
Hua 11 shoot		

Protein processing in endoplasmic reticulum		0.00018
Contig998_s_at	Heat shock protein 70	
Contig5096_at	Hypoxia upregulated 1	
Contig8758_at	UDP-glucose : glycoprotein glucosyltransferase	
Contig12326_at	Dolichyl-diphosphooligosaccharide-protein glycosyltransferase
Contig9420_at	
Contig10285_at	OST3/OST6 family protein	
Contig9432_at	Alpha 1,3-glucosidase	
Contig2576_at	Protein disulfide-isomerase A6	
PRPP biosynthesis I		0.0025
Contig5081_at	Ribose-phosphate pyrophosphokinase 4
Contig8025_at	
Jasmonic acid biosynthesis		0.0033
Contig3523_at	Enoyl-CoA hydratase/3-hydroxyacyl-CoA dehydrogenase	
Contig1737_at	Lipoxygenase1	
Contig2305_at	Lipoxygenase2	
Contig2306_s_at	Lipoxygenase2	
N-Glycan biosynthesis		0.0039
Contig12326_at	Dolichyl-diphosphooligosaccharide-protein glycosyltransferase
Contig9420_at	
Contig10285_at	OST3 and OST6 domain-containing protein	
Contig9432_at	Alpha 1,3-glucosidase	
13-LOX and 13-HPL pathway		0.0052
Contig1737_at	Lipoxygenase1	
Contig2305_at	Lipoxygenase2	
Contig2306_s_at	Lipoxygenase2	
Linoleic acid metabolism		0.0073
Contig1737_at	Lipoxygenase1	
Contig2305_at	Lipoxygenase2	
Contig2306_s_at	Lipoxygenase2	
Ribosome biogenesis in eukaryotes		0.011
Contig6911_at	Nonsense-mediated mRNA decay protein 3	
Contig3756_at	Homolog of nucleolar protein NOP56	
Contig9010_at	Exportin 1A	
Contig13525_at	Serine/threonine-protein kinase Rio1	
Contig7441_at	H/ACA ribonucleoprotein complex subunit 4	
RNA transport		0.012
Contig17611_at	Nucleoporin, Nup133/Nup155-like protein	
Contig9719_at	Nuclear pore complex protein	
Contig8398_at	Translation initiation factor eIF-3 subunit 7	
Contig8248_at	Armadillo/beta-catenin-like repeat-containing protein	
Contig6911_at	Nonsense-mediated mRNA decay protein 3	
Contig9010_at	Exportin 1A	
Plant-pathogen interaction		0.022
Contig22790_at	Coronatine-insensitive protein 1	
Contig7549_at	Calcium-binding protein CML24	
Contig14854_at	Mitogen-activated protein kinase kinase kinase 1	
Contig3262_at	Elongation factor Tu	
Contig9645_at	Putative calcium-binding protein CML21	
Contig9408_at	Chitin elicitor receptor kinase 1	
Spliceosome		0.029
Contig3732_s_at	Pre-mRNA-processing-splicing factor	
Contig998_s_at	Heat shock protein 70-4	
Contig12582_at	Pre-mRNA-splicing factor ATP-dependent RNA helicase PRP16	
Contig16451_at	DEAD-box ATP-dependent RNA helicase 21	
Contig6096_at	Mitochondrial HSO70 2	
Sphingolipid metabolism		0.038
Contig10984_at	Neutral ceramidase	
Contig6172_at	Dihydroceramidase	
Very long chain fatty acid biosynthesis		0.049
HVSMEb0010E16r2_s_at	3-ketoacyl-CoA synthase 6	
Contig3523_at	Enoyl-CoA hydratase/3-hydroxyacyl-CoA dehydrogenase	
Sucrose biosynthesis		0.049
Contig6623_s_at	Sucrose phosphate synthase 2	
Contig460_s_at	Sucrose synthase 4	

Hua 11 root		

Superpathway of polyamine biosynthesis		0.005
Contig5328_at	Agmatine deiminase	
Contig5994_s_at	Arginine decarboxylase 1	
Fatty acid biosynthesis		0.039
Contig5989_s_at	Acetyl-CoA carboxylase 1	
Indole-3-acetyl-amino acid biosynthesis		0.047
Contig12102_at	Indole-3-acetic acid-amido synthetase	
Serine biosynthesis		0.047
Contig5879_at	Phosphoserine aminotransferase	
IAA degradation IV		0.047
Contig12102_at	Indole-3-acetic acid-amido synthetase	
Phospholipid desaturation		0.047
Contig7662_at	Omega-3 fatty acid desaturase	

**Table 7:** The primers used for real-time PCR in this study.

Probe ID	Primer sequences (5′-3′)	Annotations	Fold change	
			Hua 30	Hua 11
Contig5422_at	F: CAATCCTCCAACACCAGTCA R: TCGGCAGGGACATCAAAG	Brassinosteroid insensitive 1-associated receptor kinase 1 precursor	0.8	1.15
Contig5740_at	F: GAGAATGGTAACGAGGTGG R: GGAGTTGTGTGAGCAGGGA	NAC domain-containing protein	0.15	1.15
Contig6159_at	F: CCTTCTTTCGTTTCTGCT R: GGCACTATTTCCATTACCT	PTF1	0.55	1.5
Contig7044_at	F: ACCCTCTACCACAACCGCCTCT R: ACCAACGACAGCCGCAGCA	Lung seven transmembrane receptor domain containing protein	0.05	1
Contig10690_at	F: ATCGTGTCCTCGTCGTCTTC R: AGTAGGTGGTTGCTGCTC	BZip type transcription factor bZIP1	0.15	1.55
Contig20719_at	F: ACGACGAGGACAGCGGCAACTT R: TTCACCTTGACGCCTTCTCCAC	Receptor-like protein kinase 5 precursor	0.7	4.3
HB03A08_T3_at	F: CAGATGATGGCGTTCCTCG R: TGCTCTGTGCTGCCTCCT	HSF-type DNA-binding domain containing protein	3.3	5.55

Contig13791_at	F: TCTTGGGTGATGAATGTGGTG R: AGGTTCGTGTTCCATCTC	Methyltransferase	0.55	1.2
Contig24300_at	F: ACAAACAGCATCCGAGCA R: GCCTCCAAAGTTCACATCCT	Cytokinin dehydrogenase precursor	0.9	2.4
HC02E04_T3_at	F: GAAGAAGGCGGTATGCGT R: CAGTACTTGTGAGGTAGTAGAAC	No hits	2.1	6.35

Ten salt-responsive genes in this list were selected randomly based on the microarray. The first seven pairs of primers belong to the shoot tissue, and the remaining belong to the root tissue. F: forward primer. R: reverse primer.

## Abbreviations

POD: Peroxidase
SOD: Superoxide dismutase
MAPKs: Mitogen activated protein kinases
CDPKs: Calcium dependent protein kinases
RLKs: Receptor-like kinases
CBL: Calcineurin B-like protein
CIPKs: CBL-interacting protein kinases
WAK: Wall-associated kinase
LysM RLK: Lysine motif receptor-like kinase
PP2C: Protein phosphatase 2C
ABA: Abscisic acid
JA: Jasmonic acid
GA: Gibberellin acid
ET: Ethylene
bHLH: Basic helix-loop-helix
CBF: C-repeat binding factor.
